# Study protocol to investigate biomolecular muscle profile as predictors of long-term urinary incontinence in women with gestational diabetes mellitus

**DOI:** 10.1186/s12884-020-2749-x

**Published:** 2020-02-19

**Authors:** Marilza V. C. Rudge, Fátima P. Souza, Joelcio F. Abbade, Raghavendra L. S. Hallur, João Paulo C. Marcondes, Fernanda Piculo, Gabriela Marini, Giovana Vesentini, Lehana Thabane, Steven S. Witkin, Iracema M. P. Calderon, Angélica M. P. Barbosa, The Diamater Study Group, M. V. Rudge, A. M. P. Barbosa, I. M. P. Calderon, F. P. Souza, J. F. Abbade, L. S. R. Hallur, F. Piculo, G. Marini, G. Vesentini, L. Thabane, M. S. Palma, C. F. O. Graeff, R. K. Arni, R. D. Herculano, D. F. Salvadori, S. Mateus, M. Dal Pai Silva, C. G. Magalhães, R. A. Costa, S. A. M. Lima, S. L. Felisbino, W. Barbosa, A. Atallah, M. J. B. Girão, Z. Di Bella, S. M. Uchoa, S. Payão, A. Hijas, B. Berghman, R. De Bie, L. Sobrevia, B. Junginger, F. C. B. Alves, P. S. Rossignoli, C. B. Prudencio, M. I. G. Orlandi, M. I. Gonçalves, S. K. Nunes, B. B. Catinelli, S. Quiroz, B. V. Sarmento, F. A. Pinheiro, C. I. Sartorão, R. R. Lucas, D. R. A. Reyes, S. B. C. V. Quiroz, E. M. A. Enriquez, R. G. Oliveira, J. F. Floriano, J. P. C. Marcondes, S. Barneze, T. D. Dangió, T. Pascon, P. Rossignoli, J. V. Freitas, L. Takano, F. Reis, T. D. Caldeirão, J. N. Fernandes, A. M. Carr, M. V. C. Gaitero, J. E. Corrente, H. R. C. Nunes, A. F. Candido, S. M. B. Costa, T. D. Dangió, T. Pascon, J. V. F. Melo, L. Takano, F. V. D. S. Reis, T. D. Caldeirão, A. M. Carr, G. A. Garcia, G. B. Rabadan, H. C. M. Bassin, K. S. Suyama, L. N. Damasceno, M. L. S. Takemoto, M. D. Menezes, D. G. Bussaneli, V. K. C. Nogueira, P. R. Lima, I. O. Lourenço, J. Marostica de Sá, R. A. Megid, I. P. Caruso, L. T. Rasmussen, G. M. Prata, F. Piculo, G. Vesentini, M. A. Arantes, G. A. R. Ferraz, L. P. Camargo, M. R. Kron, J. E. Corrente, H. R. C. Nunes

**Affiliations:** 1 Department of Gynecology and Obstetrics, Botucatu Medical School (FMB), São Paulo State University (UNESP), CEP18618-687, Sao Paulo, Brazil; 2 Physics Department, Institute of Biosciences, Letters and Exact Sciences, Multiuser Center for Biomolecular Innovation, UNESP-São Paulo State University, Sao Paulo, Brazil; 3 Physiotherapy Department, Faculdades Integradas de Bauru, FIB, Sao Paulo, Brazil; 4 Universidade do Sagrado Coração (USC), Jardim Brasil, Bauru, Sao Paulo Brazil; 5 Department of Health Research Methods, Evidence, and Impact, McMaster University, Hamilton, ON Canada; 6 Biostatistics Unit, Father Sean O’Sullivan Research Centre, St Joseph’s Healthcare-Hamilton, Hamilton, ON Canada; 7 Department of Obstetrics and Gynecology, Weill Cornell Medicine, New York, NY USA; 8 Institute of Tropical Medicine, University of Sao Paulo Medical School, Sao Paulo, Brazil; 9 School of Philosophy and Sciences, Department of Physiotherapy and Occupational Therapy, UNESP-São Paulo State University, Marília, Sao Paulo Brazil

**Keywords:** Gestational diabetes mellitus, Hyperglycemic myopathy, Pelvic floor muscles, Rectus abdominis muscles, Urinary incontinence, Proteomics, Collagen, Electromyography, Transmission electron microscopy

## Abstract

**Background:**

Pelvic floor muscles (PFM) and rectus abdominis muscles (RAM) of pregnant diabetic rats exhibit atrophy, co-localization of fast and slow fibers and an increased collagen type I/III ratio. However, the role of similar PFM or RAM hyperglycemic-related myopathy in women with gestational diabetes mellitus (GDM) remains poorly investigated. This study aims to assess the frequency of pelvic floor muscle disorders and pregnancy-specific urinary incontinence (PS-UI) 12 months after the Cesarean (C) section in women with GDM. Specifically, differences in PFM/RAM hyperglycemic myopathy will be evaluated.

**Methods:**

The Diamater is an ongoing cohort study of four groups of 59 pregnant women each from the Perinatal Diabetes Research Centre (PDRC), Botucatu Medical School (FMB)-UNESP (São Paulo State University), Brazil. Diagnosis of GDM and PS-UI will be made at 24–26 weeks, with a follow-up at 34–38 weeks of gestation. Inclusion in the study will occur at the time of C-section, and patients will be followed at 24–48 h, 6 weeks and 6 and 12 months postpartum. Study groups will be classified as (1) GDM plus PS-UI; (2) GDM without PS-UI; (3) Non-GDM plus PS-UI; and (4) Non-GDM without PS-UI. We will analyze relationships between GDM, PS-UI and hyperglycemic myopathy at 12 months after C-section. The mediator variables to be evaluated include digital palpation, vaginal squeeze pressure, 3D pelvic floor ultrasound, and 3D RAM ultrasound. RAM samples obtained during C-section will be analyzed for ex-vivo contractility, morphological, molecular and OMICS profiles to further characterize the hyperglycemic myopathy. Additional variables to be evaluated include maternal age, socioeconomic status, educational level, ethnicity, body mass index, weight gain during pregnancy, quality of glycemic control and insulin therapy.

**Discussion:**

To our knowledge, this will be the first study to provide data on the prevalence of PS-UI and RAM and PFM physical and biomolecular muscle profiles after C-section in mothers with GDM. The longitudinal design allows for the assessment of cause-effect relationships between GDM, PS-UI, and PFMs and RAMs myopathy. The findings may reveal previously undetermined consequences of GDM.

## Background

Gestational diabetes mellitus (GDM) and urinary incontinence (UI) are two clinical entities with substantial social and economic burden, associated with significant direct and indirect public health costs [1]. In addition, they often affect women at a young age. A new emphasis in the early diagnosis of GDM aims not only to prevent perinatal morbidity and mortality but also to identify potential long-term maternal complications [2]. In 2013, 382 million people had diabetes, and there is a strong likelihood that this will rise to 592 million by 2035. Brazil, a low-and-middle-income country, is one of the top 10 countries in terms of numbers of diabetic people, with projections of almost 19.2 million [3].

Beginning in 2008 [4] researchers reported that GDM resulted in postpartum genitourinary dysfunction that persisted for 2 years after delivery [5, 6]. Previous GDM was shown to be an independent risk factor for Pregnancy Specific- Urinary Incontinence (PS-UI), a risk factor for UI two-years after the Cesarean (C) section. The link between GDM and PS-UI with increased long-term UI highlighted the potential under-explored contribution of GDM to the mechanisms leading to long-term UI [5]. This was an unanticipated finding that was not included in the classical definition of long-term GDM-related outcomes [2]. Thus, to improve the quality of life in women with GDM, it is imperative to understand the connection between GDM and PS-UI. Delineation of the involved mechanism will lead to the development of protocols to reduce long-term UI complications.

**Fig. 1:**
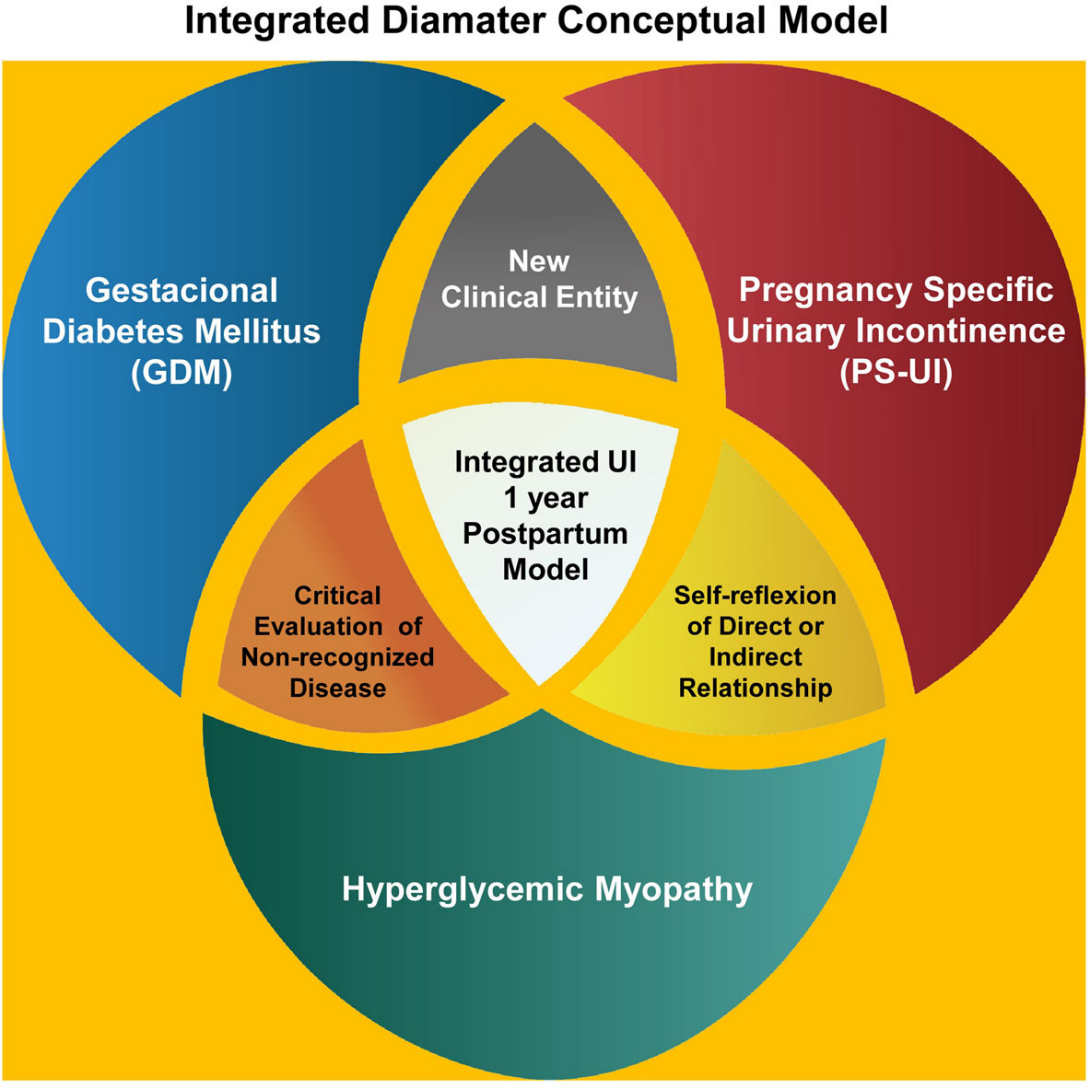
Conceptual model of the role of integration between GDM, PS-UI and RAM-PFM myopathy as a new triad in determining the prevalence of long-term maternal UI

The conceptual model delineated by Delancey et al. in the Lifespan Analysis of pelvic floor muscles (PFM) [7] identified GDM as an inciting factor for long-term UI [8]. GDM and long-term UI are two clinical entities with a substantial economic and health impact, ethical concerns related to the use of PFM biopsy material for investigation have limited clinical investigation in this area. Utilizing our expertise in diabetic pregnant rat models [9–14], we initiated a “*bedside to bench”* translational study to obtain a deeper understanding of the influence of GDM and pregnancy on urethral and rectal myopathology. The conceptual model is shown in Fig. 1.

### Relevance and uniqueness of our translational studies: from “bedside to bench”

Presently, human studies are based on clinical findings of a high incidence of UI 2 years after GDM. The role of GDM in the pathophysiological development of UI remains unclarified [15]. Investigations linking both entities with the synergistic co-activity of the PFM and RAM are insufficient [16]. Current clinical studies have provided evidence for PFM involvement in the etiology of PS-UI [17–20], including more recently, the contribution of extracellular matrix (ECM) components, such as collagen [21] and glycosaminoglycans (GAGs) [22] to female pelvic floor function.

Pregnancy itself alters the PFM, in preparation for parturition. Alperin et al. reported that adaptations during pregnancy occur in the myofibers of rat PFMs, which increase in length by the serial addition of sarcomeres [23]. Although the biochemical alterations in PFM extracellular matrix due to pregnancy include an increase in collagen V and a differential response in enzymatic vs. glycosylated collagen crosslinks, the relationships between PFM biochemical and mechanical parameters remain unclear [24].

In pregnant diabetic rats, our research group identified thinning, atrophy, disorganization, and disruption in the circular annulus associated with the co-localization of fast and slow fibers. This was accompanied by a steady decrease in the proportion of fast versus slow fibers (fast: slow, 1.5:1) associated with increased collagen deposition, severe fibrosis, lipid droplets, and numerous subsarcolemmal, and intermyofibrillar mitochondria [25]. These characteristics differentiated the urethral striated muscles of short-term severe diabetic pregnant rats and long-term mild diabetic pregnant rats from virgin, pregnant and diabetic control groups [20]. The diabetic models were obtained by streptozotocin injection, and according to the dose and duration of the hyperglycemic stimulus, they are classified as short-term severe diabetes or long-term mild diabetes [25, 26]. The increase in collagen and severe fibrosis may also affect the contractile properties of the urethra [26, 27]. The ECM molecular analyses of urethral components showed no difference in type I and type III collagen expression levels in short-term severe diabetic pregnant rats. However, the collagen type I /III ratio was higher in the hyperglycemic pregnant rats compared to the virgin group. The increased collagen type I/III ratio can favour a stiffer urethral tissue leading to an influence of hyperglycemia on the remodelling of urethral connective tissue in pregnant diabetic rats [28, 29].

In conclusion, these experimental results demonstrated that both long-term mild hyperglycemia and short-term severe hyperglycemia negatively impacted the muscle health in rats. RAM is subjected to similar structural changes related to pregnant diabetic myopathy [25, 26, 28] with intramuscular transformation and reorganization in fiber types [30–32].

Liu et al. showed that diabetes-induced time-dependent alterations of the external urethral sphincter and abnormal activity presumably contributes to the abnormal voiding function seen in diabetes [33]. Obesity, insulin resistance, and type 2 diabetes mellitus (T2DM) are associated with a significantly increased prevalence of vascular fibrosis and stiffness [34].

### Significance of the proposed study: the bench-bedside proposal

Pregnant women with GDM are at increased risk of UI [35]. However, the direct association between women with GDM and PS-UI is undetermined. A few studies have reported women with GDM to have a high prevalence of UI due to higher body mass index, obesity, and macrosomia in their infants [36, 37]. Although the exact mechanism remains unclear, excess weight gain during pregnancy may exert pressure on the PFM, which increases pressure on the bladder and influences urethral mobility, leading to UI [38]. In addition, hyperglycemia in women with GDM may cause polyuria or detrusor instability. In a cross-sectional survey, Kim et al. [4] examined the prevalence of PS-UI among women who had GDM during pregnancy, surveying 228 women, and found that 49% of women had weekly or more frequent incontinence during pregnancy, and 50% of the women reported PS-UI within 5 years following delivery. A high prevalence of obesity may have contributed to the elevated rate of PS-UI during pregnancy and after delivery. Obesity was present in 42% of women during pregnancy and in 46% after delivery. Their conclusion was that PS-UI was common among women with GDM.

Barbosa et al. [5] and Chuang et al. [6] determined whether GDM was an independent risk factor for PS-UI postpartum. They found that women with GDM exhibited symptoms of PS-UI for up to 2 years postpartum in comparison to women without GDM. They concluded that GDM was an independent risk factor for UI postpartum with a significant impact on the quality of life. They also suggested that women with GDM should be provided with timely consultation and support once UI occurs.

Factors associated with PS-UI, before or at the time of GDM diagnosis, have also been associated with UI two-years post-C-section. Having GDM in a previous pregnancy was an independent risk factor for PS-UI [5]. PS-UI is a new clinical entity linked to GDM with a direct or indirect relationship to hyperglycemic myopathy. The possibility that it may represent the first clinical symptom of PS-UI requires more investigation. Experimental results on urethral muscles and RAM from pregnant diabetic rats have revealed a variety of abnormalities in muscles involved in urinary continence. The abnormalities in skeletal muscles and ECM in pregnant diabetic rats provide a rationale for multidisciplinary human in vivo research to investigate similar hyperglycaemic myopathy in GDM skeletal muscles during C-section. Brazil, a low-middle income country with a high prevalence of GDM and T2DM has established a Perinatal Diabetes Research Center (PDRC) at Botucatu University Hospital. The Center provides the initial GDM diagnosis and maintains strict glycemic control during pregnancy. The collection of muscle samples for in-depth analysis of the connection between GDM history and PS-UI with long-term UI has not previously been attempted.

Findings from GDM, PS-UI, and RAM/PFM muscle tissue lesions will be integrated to predict long-term risk for UI. Ethical approval was obtained to initiate RAM sample collection during C-section at PDRC, due to successful results on our combined clinical and experimental studies described above.

### Potential mediators and moderators of GDM and UI

We seek to attempt to clarify the mechanisms that link two highly prevalent illnesses that affect women’s health, are associated with high health costs, and are expected to increase in frequency.

Two procedures to evaluate underlying/influential factors are the mediator and moderator variables [39, 40]. The mediator model assumes that predictor variables are responsible for changes in mediator variables, and that mediator variables subsequently cause changes in outcome variables [41]. The moderator model analyzes qualitative or quantitative variables that affect the magnitude of the relation between an independent and a dependent variable [42].

Despite the established inter-relationship between DM and UI and pregnancy and UI, the inter-relationships between GDM and long-term UI have rarely been studied using a mediator or moderator model [39].

**Fig. 2:**
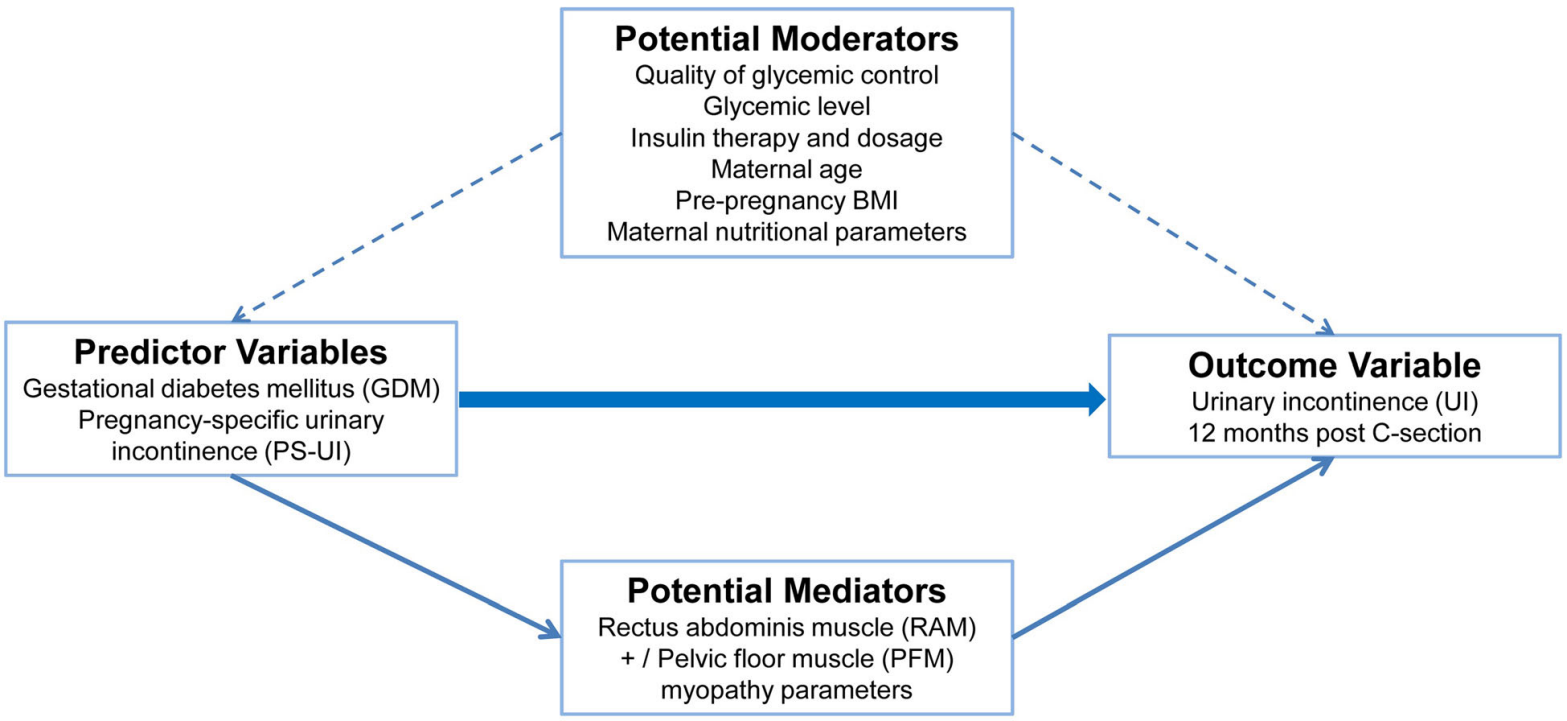
Potential confounders –Mediators and Moderators of Diamater study

Furthermore, little information has been reported concerning a positive correlation between GDM and long-term UI [5, 35, 39, 42–45]. An outline of our proposed analysis is described in Fig. 2. It has components similar to prior investigations [40, 46].

The longitudinal assessment of the role of GDM plus PS-UI on UI 12 months after C-section using mediator variables provides a foundation for optimizing future treatment for individual women and under what circumstances [47, 48].

### GDM plus PS-UI and clinical-biomolecular muscle profile as a long-term predictor of UI

GDM during pregnancy and its association with PS-UI and a specific PFM or RAM biomolecular muscle profile constitute a new triad leading to long-term UI after C-section. We hypothesize that UI 12 months after C-section will be higher in women who developed GDM plus PS-UI compared to women without these factors. Our secondary hypotheses are: (1) GDM plus PS-UI is an independent risk factor for UI development 12 months after C-section and (2) Hyperglycemic-related myopathy parameters are potential mediators of the association between GDM plus PS-UI and symptomatic UI 12 months after C-section.

Therefore, we aim to quantify the clinical-biomolecular muscle profile of PFM and RAM hyperglycemic myopathy during pregnancy in GDM women with PS-UI as exposure, on UI 12 months after C-section and compare the profile to that obtained from women who did not have GDM or PS-UI. We include an analysis of the mediators and moderator variables that are related to PFMD and UI in this evaluation (Fig. 2).

The primary and secondary research questions to be addressed in this research are:

#### Primary question

- ➣ Is the incidence of UI 12 months after C-section different in women who developed GDM plus PS-UI compared to women without these conditions?

#### Secondary questions

- ➣ Does the presence of both GDM plus PS-UI modify the risk of UI 12 months after C-section independently of the moderator variables?
- ➣ Which mediator variables of hyperglycemic myopathy are abnormal in women who developed GDM and PS-UI compared to women without these conditions?

### Aim of the study

- ➣ To analyze moderators and mediators involved in the relationship between GDM plus PS-UI and UI 12 months after C-section.
- ➣ To assess the association between GDM plus PS-UI and clinical assessment of PFM and RAM by (1) utilization of ultrasonographic, electromyographic and morphological assays, (2) molecular analysis of muscle fiber function, ECM-related genes and proteins, gene and protein expression, (3) OMICS and (4) ex-vivo assessment of RAM contractility. This will result in a comprehensive characterization of the clinical-biomolecular muscle profile of PFM and RAM hyperglycemic myopathy.
- ➣ To determine the correlation between maternal age, pre-pregnancy body mass index, nutritional parameters, glycemic level, quality of glycemic control, insulin therapy, and insulin dose with GDM plus PS-UI and UI 12 months after C-section.
- ➣ To examine the unidirectional relationship among the triad GDM, PS-UI, and hyperglycemic myopathy in UI 12 months after C-section compared with the normal population (Normoglycemic without PS-UI and without hyperglycemic myopathy).

## Methods

### Study area, design, and setting

The Diamater is an ongoing cohort prospective study of GDM pregnant women with PSUI following C-section, seen at the Perinatal Diabetes Research Center (PDRC), a tertiary center for Diabetes Care in Brazil, from April 2017 to March 2022. PDRC is located in three different cities of São Paulo State, including PDRC, FMB-UNESP, Botucatu, Clinic of Children’s Hospital Maternal Risk Pregnancy, Faculdade de Medicina de Marília (FAMEMA)-UNESP, Marilia and Regional Hospital-UNESP of Assis.

### Participants

Participants need to satisfy the following inclusion and exclusion criteria.

### Eligibility criteria

#### Inclusion criteria

Eligible participants are volunteer pregnant women with a GDM diagnosis between 24 and 28 weeks of pregnancy who have follow-up prenatal care at PDRC and C-section delivery. Subjects will be primigravida or second gravida women who underwent a planned C-section in their previous pregnancy, between 18 to 40 years old; with a singleton pregnancy and seen between February 2017 and March 2022.

#### Exclusion criteria

Pregnant women aged < 18 years and > 40 years old, pre-pregnancy UI, known type 1 or type 2 diabetes mellitus, preterm delivery (< 37 weeks of gestation), multiple pregnancies, chronic arterial hypertension, pre-eclampsia, eclampsia, known fetal anomaly, urogenital cancer, high-risk pregnancy, multiple sclerosis, asthma, chronic sinusitis, ulcerative colitis, biliary cirrhosis, inflammatory bowel disease and/or anorectal surgery/ personal history of connective tissue disease, vaginal delivery or any clinical condition that may have jeopardized health status or the ability to provide written informed consent.

### Method of recruitment of participants

Participants will be recruited in two phases. The objective of the first phase (Phase 1) is to identify normoglycemic or hyperglycemic pregnant women with or without PS-UI. These women will be followed up until delivery. The second phase (Phase 2) of the study will begin at the time of delivery (C-section), and these women will be followed for up to one-year postpartum.

#### Phase 1

Pregnant women will be recruited at the time of antepartum testing for GDM at 24 to 28 weeks gestation. The study will be described, and the women will be asked to volunteer and sign the informed consent form. Socio-economic status information will be collected. Clinical data and blood samples will be collected, questionnaires about UI will be applied, and clinical PFM and RAM assessment will be performed. At 36 weeks of pregnancy, another informed consent will be requested to collect the RAM sample (during the C-section) for OMICS, genetic, protein, and morphological analyses (Additional file 1). The criteria for performing a C-section will be established by the hospital staff. The women will be included in phase 2 only if they have a cesarean delivery for obstetrical or clinical indication.

#### Phase 2

Women will be included in phase 2 cohort following C-section delivery. The cohort groups for the primary outcome will be established at this point. Prenatal care assistance, including GDM diagnosis and treatment, as well as the PS-UI diagnosis made at any time during pregnancy, will be used in the composition of the four study groups.

### Follow-up

All recruited patients will be evaluated as follows: visit − 2- at 34 to 38 weeks gestational age (T2) and inclusion in the C-section delivery cohort (beginning of phase 2). After visit − 3-within 24 and 48 h postpartum (T3), visit − 4- 6 weeks postpartum (T4), and 6 and 12 months postpartum (T5 and T6, respectively). Four cohorts will be followed-up based on the results of GDM and PS-UI diagnosis: (i) GDM plus PS-UI; (ii) GDM without PS-UI; (iii) Non-GDM plus PS-UI and (iv) Non-GDM without PS-UI.

### Variables

#### GDM diagnosis

The diagnostic protocol for GDM is a 75-g oral glucose tolerance test (OGTT) (Table 1) combined with the glycemic profile in use at PDRC-UNESP-Brazil since 1984 [46] for pregnant women without confirmed GDM or overt diabetes in their first trimester of pregnancy. All hyperglycemic pregnant women will be classified as GDM.

**Table 1:** GDM diagnosis by 75 g-OGTT and glycemic profile

	Fasting plasma glucose	5.1 mmol/L	92 mg/dL
75 g-OGTT	1-h	10.0 mmol/L	180 mg/dL
	2-h	8.5 mmol/L	153 mg/mL
Glycemic Profile	Fasting plasma glucose	5.1 mmol/L	92 mg/dL

#### Prenatal management of patients with GDM

All hyperglycemic pregnant women will undergo the same treatment, including adequate nutritional advice from nutritionists, encouragement to exercise regularly, and insulin associated with dietary advice if necessary [46].

#### Diagnosis of UI and definition of PS-UI

For the primary outcome of the study, UI will be considered if there is any involuntary leakage of urine according to the International Continence Society definition of UI [49]. PS-UI is defined as any urinary leakage newly onset during pregnancy ascertained by self-reporting [50]. Participants will be asked whether before pregnancy they had leaked or lost control of even a small amount of urine with activity such as coughing, lifting, or exercise or if they experienced an urge or pressure to urinate and could not go to the toilet fast enough (urge UI). Pregnant women with PS-UI and/or urge UI before pregnancy will be excluded from the study.

Pregnant women who reported PS-UI will be asked to complete the Brazilian version of the International Consultation on Incontinence Questionnaire-Urinary Incontinence-Short Form (ICIQ-SF) [51]. The participants will also be asked about the frequency and quantity of urinary leakage to assess the validated Incontinence Severity Index (ISI). The severity will be further graded depending on the frequency of leakage (grade I: once a week at most; grade II: two or more episodes per week, but not daily and grade III: one or more episodes of leakage per day) according to ISI questionnaire [52].

#### Assessment procedures and instrumentation

**Fig. 3:**
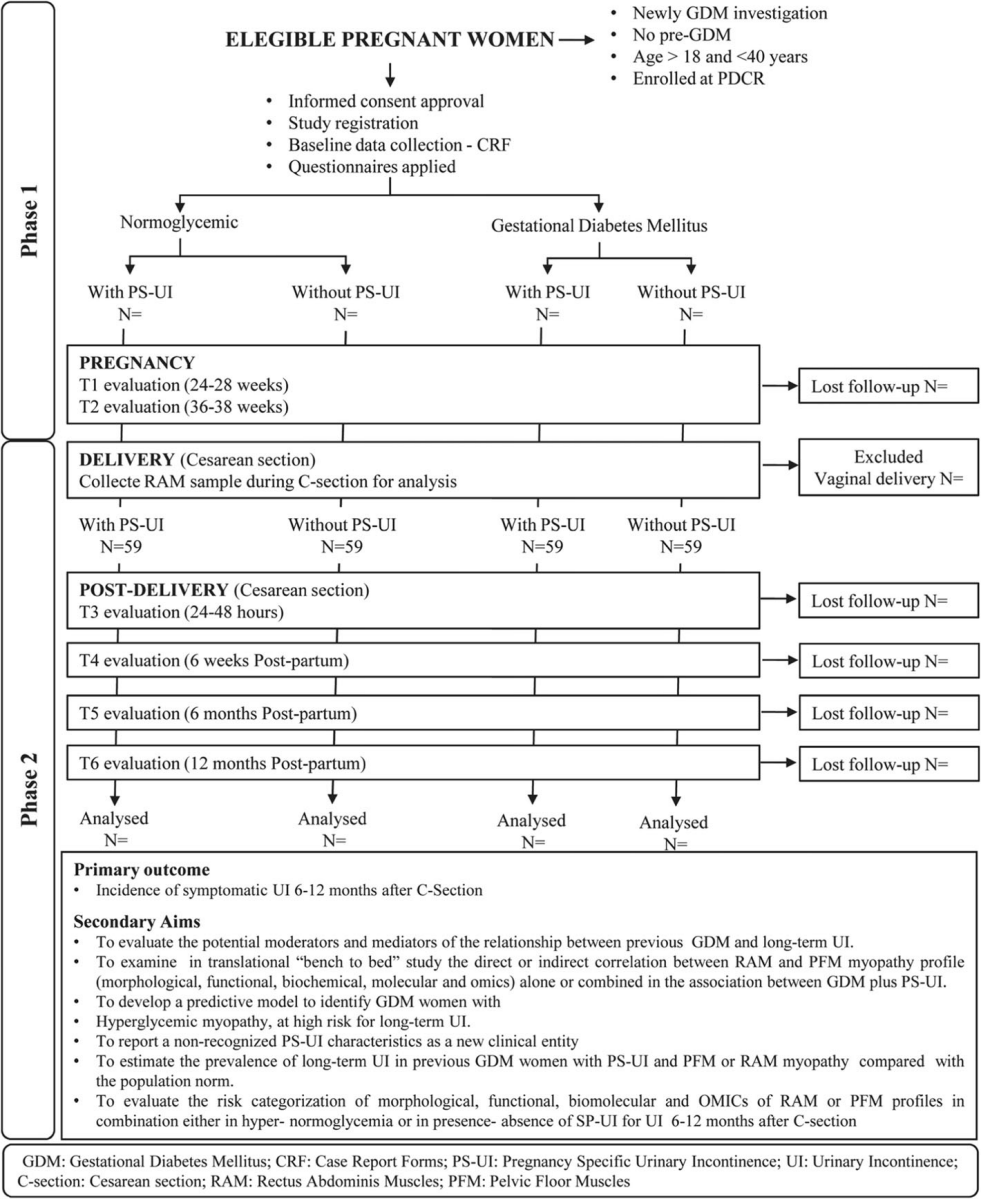
Phases and Follow-up timelines of the proposed protocol

A comprehensive questionnaire was developed for collecting information on the socio-demographic, anthropometric, medical, and obstetrical history. A Case Report Forms (CRF) will be established for data collection of variables for the Diamater study from all subjects. In summary, the databank includes patients’ identification, socio-economic status, demographic characteristics, obstetric history, delivery and birth outcomes, perinatal complications, neonatal morbidity, maternal postpartum follow-up and all variables related to biochemical and molecular analysis, functional and morphological assessments during pregnancy and at 6 weeks and 6–12 months after C-section (Table 2). Additional information will be collected to describe the study population. Several variables will be assessed as confounders or effect modifiers of the relationship between GDM and/or PSUI and intestinal microbiota colonization and UI 12 months after C-section. The participant flow chart is shown in Fig. 3.

**Table 2:** Data collection questionnaire, clinical assessment and laboratory analysis of study participants

	Pregnancy		Delivery	Post-partum		
	T1	T2	T3	T4	T5	T6
	24-28th weeks	36-40th weeks	C-Section	6 weeks	6 months	12 months
Socioeconomic status						
Address/phone number/social media	x	x	x	–	x	x
Age	x	x	x	–	x	x
Occupation	x	x	x	–	x	x
Ethnicity	x	x	x	–	x	x
Religion	x	x	x	–	x	x
Marital status	x	x	x	–	x	x
Education level/background	x	x	x	–	x	x
Occupation	x	x	x	–	x	x
Smoking/drinking/illicit drug use	x	x	x	–	x	x
Clinical data						
Infections, chronic diseases, family disease history, smoking/drinking, comorbidities and surgeries	x	x	x	–	x	x
Questionnaires						
Diagnosis of PS-UI (ISI and ICIQ)	x	x	–	–	–	–
Diagnosis of UI (ISI and ICIQ)	–	–	–	x	x	x
Newborn data						
Weight	–	–	x	–	–	–
Length	–	–	x	–	–	–
Ponderal index	–	–	x	–	–	–
Apgar score	–	–	x	–	–	–
Complications during gestation and/or delivery	–	–	x	–	–	–
Obstetrics data						
Gestational age	x	x	x	–	–	–
Height	x	–	–	–	–	–
Pre-gestational weight	x	–	–	–	–	–
Current weight	x	x	x	x	x	x
Weight gain during pregnancy	x	x	x	–	–	–
Clinical complications during delivery	–	–	x	–	–	–
Biochemical, Molecular, Functional and Morphological assessments						
A) Laboratory assessments						
Glucose, Cortisol, Insulin, PTH, Calcitonin, Phosphorous, Potassium, Calcium, Sodium, Magnesium, Vitamin A and Vitamin D	x	x	x	x	x	x
B) Physical assessments						
PFM						
Digital palpation	x	x	–	x	x	x
US	x	x	–	x	x	x
EMG	x	x	–	x	x	x
RAM						
US	x	x	–	x	x	x
EMG	x	x	–	x	x	x
C) RAM sampling RAM						
OMICS (Transcriptomics, Metabolomics, and Proteomics)	–	–	x	–	–	–
Expression of muscle fibers and EM-related genes and proteins	–	–	x	–	–	–
Ex-vivo contractility	–	–	x	–	–	–
Morphological analysis	–	–	x	–	–	–

#### Moderator variables

The moderator variables to be included in the analysis are maternal age, socio-economic status, educational level, ethnicity, pre-pregnancy body mass index [53], weight gain during the pregnancy [54], maternal nutritional parameters, quality of glycemic control, glycemic level during pregnancy and insulin therapy and dose.

#### Mediator variables

Clinical assessment of PFM and RAM, the RAM sample collection and laboratory assays will be included in this analysis to characterize the hyperglycemic myopathy. All specific protocols for clinical assessment and laboratory analysis of pelvic floor muscles and rectus abdominis muscle as mediator variables are described in Additional file 1.

#### Predictor variables

GDM and PS-UI are considered as predictor variables.

#### Outcome variables

PFM and UI 12 months after C-section are considered the outcome variables. The PFM and RAM analysis will be considered as mediator variables in this interrelationship. The common procedure for this is to apply statistical methods on production data to establish the interrelationship. The detailed procedure is described in Additional file 1.

### Data measurement

#### Digital palpation and vaginal squeeze pressure

For the digital vaginal evaluation, the examiner will follow the Messelink et al. orientation [55]. A perineometer value higher than 33.6 mmHg will be considered as normal based on our previous nulliparous women study [5].

#### 3D pelvic floor ultrasound (PFUS)

Levator function will be assessed using the modified Oxford grading of muscle strength scoring system [56]. Transperineal ultrasonographic assessment biometry of PFM and RAM data will be collected, including anteroposterior diameter, transversal diameter, Hiatal area, and levator ani muscle thickness, at rest, contraction and Valsalva maneuver. AGE Voluson “i” system with RAB 2–6 RS (2–6 MHz) curved array three-dimensional transducer (GE Healthcare, Zipf, Austria) with an 8–4 MHz curved-array volume transducer at an acquisition angle of 70° in the sagittal plane and 85° in the coronal plane (frame rate is approximate 3 Hz) will be used [57, 58].

#### 3D RAM ultrasound protocol

First, the transducer will be placed above the umbilicus. It will then be moved laterally from the midline until the cross-section of the muscle will center on the image [59]. Ultrasound images will be acquired in the B-mode using a portable ultrasound unit, the GE Voluson “i” system with a RAB 2–6 RS (2–6 MHz) curved array three-dimensional transducer (GE Healthcare, Zipf, Austria) [59]. The thickness of the lateral abdominal muscles will be assessed at rest and during submaximal contraction of these muscles, bilaterally analyzing 36 images for each participant [60]. The anatomic and functional evaluation will be conducted in the lower right portion of RAM in a transversal direction along the muscle [56]. Before RAM image acquisition under PFM contraction, the patient will undergo a PFM function test according to the International Continence Society [55] to avoid bias during US evaluation, and to analyze its function subjectively.

#### Electromyography

Surface electromyograms (EMGs) will be recorded using an eight-channel electromyography (EMG) device (New MiotoolUro Wireless; Porto Alegre, Brazil), 16-bit A/D converter, a sampling frequency rate fixed at 2000 Hz, automatic gain, safety isolation for 3000 V and common moderate (CMRR) of 126 dB. The signal will be filtered using a 20–500 Hz dual-pass Butterworth second-order digital filter. The preparation of the skin, sensor placement, and location preparation will be acquired according to the recommendation of the Surface Electromyography for the Non-Invasive Assessment of Muscles (SENIAM). Modified Glazer protocol will be used to verify muscle activity during rest-activity and fast and hold contractions. These protocols will be used twice, one only asking to increase abdominal pressure without conscious contraction of the pelvic floor and the second time only performing pelvic floor contractions.

### Laboratory data of maternal blood collection and analysis for biochemical analysis

Serum levels of relaxin, insulin, glucose, chemokine (C-C motif) ligand 7 (CCL7), calcium, calcitonin, parathyroid hormone (PTH), and vitamin D in maternal blood will be determined. The serum aliquots will be stored at − 80 °C for further batch processing according to the specific assay kit (relaxin: R & D Systems Catalog Number DRL200; Insulin: R&D Systems Catalog Number DINS00; and CCL7; R&D Systems Catalog Number DCC700). The total calcium levels (Catalog No. 448), calcitonin and PTH levels by the chemiluminescence method, vitamin D by high-performance liquid chromatography (HPLC) by following the procedures of processing and analysis of the manufacturer’s manual.

### RAM sample collection, storage, and laboratory assays

#### RAM sample collection

RAM samples will be collected according to a specific protocol. Morphological characterization, molecular analysis of muscle fiber function, ECM-related genes and proteins, gene and protein expression, OMICS, and ex-vivo assessment of RAM contractility will be performed according to specific protocols to characterize the hyperglycemic myopathy.

A biological sample of RAM from the region of a transverse Pfannenstiel incision will be obtained during C-section. RAMs are of mixed muscle fiber type [61, 62]. A 4 cm RAM tissue will be sampled in the lower portion. Dissections of skeletal muscle will be obtained within 10 min of delivery, dissected free from visible adipose and connective tissues, immediately divided into six fragments and stored according to different assays for further analysis.

#### Morphological assays

Part of the RAM will be harvested, powdered with talcum, frozen in liquid nitrogen and maintained at − 80 °C until processed by histochemical and immunohistochemical techniques for morphometric analysis and, to analyze the distribution, quantification, and characterization of key structural ECM components, such as type I and III collagens, the collagen type I/III ratio and GAGs. It will be examined via light microscopy and photographed. The morphometric analyses will be performed with Image-Pro Plus software (Version 7.0, Media Cybernetics, Silver Spring, MD) at Case Western Reserve University (OH). The proportions of RAM that fit each of the following types will be recorded: striated muscle, collagen, and blood vessel. Other samples (three samples/group) will be immersed in a fixative solution containing 0.1% ruthenium red, 3% glutaraldehyde, and 0.1 M cacodylate buffer for 12 h at 4 °C before transmission electron microscopy for ultrastructural analysis.

### Sample size

It is not possible to calculate the sample size for Phase 1 of the recruitment. All eligible pregnant women will be invited to be included in this phase. Considering that the incidence of UI was higher at 24-months in GDM-positive C-section post-partum women (44.8%) as compared with women without GDM (18.4%) [5], assuming a two-sided confidence level of 95% and power of 80%, it will be necessary to follow-up 49 C-section post-partum women in each purposed cohort to identify at least a 22% difference between them. Considering a 20% loss in follow-up, we will include 59 women in each cohort, which means that 236 women will be included in this research.

### Statistical analysis

The mediator models assume that the predictor variable causes changes in the mediator variable, and the mediator variable then causes changes in the outcome variable. Mediation analysis will be conducted to evaluate whether the effect of GDM plus PS-UI on long-term UI is mediated through alteration in levels of all assessed parameters. The patient flow will be summarised using a diagram while social-demographic and obstetrics baseline characteristics will be reported as mean and standard deviation for continuous variables and count for categorical variables.

Multivariate logistic regression analyses will be conducted to identify the mediator variables and the outcome (UI 12-month C-section post-partum) that will be changed by the predictor variable (GDM plus PS-UI) as an independent variable after adjusting for confounders. Clinically important covariates will be investigated as potential confounders whenever appropriate for each outcome. There is likely to be missing data that will likely with the duration of the follow-up. The multiple imputations will be used to handle missing data [63]. Results will be expressed as odds ratio [OR], 95% confidence intervals and associated *p*-values. For all tests, the level of significance will be set at a p-value < 0.05. Assessment of the functional, morphological, biochemical, and molecular mediator variables are presented in Table 3.

**Table 3:** Statistical tools will be used for the study

	Variables	Statistical methods
Clinical data		
Incidence of UI 12-month post-partum	Categorical	Multivariate analysis, odds ratio and 95% of the Confidence interval
ISI Questionnaire	Categorical	Chi-square test with corrections
PFM: Vaginal muscle contraction pressure; digital vaginal evaluation and US	Categorical	Chi-square test with corrections
RAM: US and Electromyography	Categorical	Chi-square test with corrections
Laboratorial data		
Comparison of serum concentration of CCL7; relaxin; calcium; parathyroid hormone; calcitonin; Vitamin D and Insulin	Continuous	Repeated Measures t-test or one-way analysis of variance (ANOVA) and Area under the curve
Laboratory assays of RAM sample		
Morphological assay Pathological, morphometric, immunohistochemical and ultrastructural	Continuous	ANOVA followed by Tukey’s multiple comparison tests for the normally distributed variables
	Categorical	Chi-square test with corrections
Gene expression (PCR arrays®)	Continuous	ANOVA followed by Tukey’s multiple comparison tests for the normally distributed variables
Protein expression (Western blot)	Continuous	Student’s t-test and comparison of multiple means used an ANOVA with a Tukey post-test to compare two variables where applicable
Transcriptomics, Metabolomics and Proteomics	–	Previously described in the text
Ex-vivo assessment of RAM Contractility	Continuous	Student’s t-test and comparison of multiple means used an ANOVA with a Tukey post-test to compare two variables where applicable

OMICS statistical analysis

For transcriptomics, the bioinformatics analysis will be done using the software fastqc63, FASTQ Quality Filter, FASTA/Q Clipper to identify and remove the low-quality adapters and reads. The RNA reads will be aligned to the reference sequences using the TopHat2 and Bowtie2 software. The relative gene expression will be quantified by Deseq algorithm, evaluated in Bioconductor/R64 packages. Genes/sequences with padj< 0.05 and log2 fold change > 2 or < −2 will be considered for subsequent analysis

The multivariate analyses of the M-NMR data will be carried out by PCA (Principal Component Analysis) or multivariate methods of supervised statistics (PLS or OPLS), aiming to build models for Brazil of the samples and thus Extract metabolic signatures from specific groups [64]. The calculations will be made by written (homemade) programs by our research group using the MATLAB platform (MathWorks, USA). The scoring and loading charts of the PCA analysis will be used for the display of the data. In the score graph, each point represents an NMR spectrum (i.e. a sample) for one of the main components, and the loading graph visualizes the contribution of key metabolites (statistically significant variables) to the Main component. The PCA method will be applied initially to the complete set of data, 11 to which this realization a form of unsupervised analysis. Then the multivariate supervised methods (PLS or OPLS) will be employed with a prior differentiation of the sampling groups

### Patient and public involvement

The development of the research questions and outcome measures was performed based on previously published clinical research and from our prior translational studies. Patients and the public will not be involved in the study design, recruitment, and the conduct of the study. The patients involved in this study are those admitted to the University Hospital-Botucatu Medical School-UNESP. After completion of the data analysis, we plan to introduce the results into our routine practice of long-term follow-up care. We are also planning to translate the results into short and easy-to-read summaries to disseminate it to the relevant patient community groups through local media.

### Ethics and dissemination

The Institutional Review Board of Botucatu Medical School-UNESP (CAAE: 82225617.0.0000.5411) approved the Diamater study protocol. Written informed consent will be obtained from all subjects, and the procedures used in the study are in accordance with the guidelines of the Helsinki Declaration on human experimentation.

The patients will be exposed to only low risk during the study. The participants’ information will be stored electronically or in a locked file in the project office. The documentation of research data and management of the study will follow the Guideline for Good Clinical Practice in collecting, storing, and processing data anonymously [50]. All samples will be managed and stored in a specific biorepository for the research and results disseminated in peer-reviewed journals.

### The potential benefit to subjects

While there is no direct benefit to subjects, being asked about their health status in the first year postpartum by a member of the research team every 3 months may raise the mother’s awareness of her health and that of her newborn. At PDRC, during the follow-up period, a nutritional assessment and exercise programs will be offered as well as long-term health education and promotion that is crucial to effectively control GDM and UI. This is particularly important for pregnant women in a low and middle –income country like Brazil.

The study team will also focus on expanding the number of new researchers through mentorship and supervision. A longitudinal study, such as the Diamater study protocol offers undergraduate, masters, Ph.D and postdoctoral students a special environment to gain experience with longitudinal data sets. Based on this premise, scholarships for these students are included in the financial support to Diamater study from our state and national research agencies. This prospective study is intended to create and reinforce national and international cooperation, as well as to attract international postdoctoral students and provide new biomarkers that will improve the ability to identify risk factors in GDM women more accurately.

### Ethics and dissemination

The primary investigator has extensive training and experience in clinical research and relevant bioethics. The research staff will include a team of primary researchers that have extensive qualifications and expertise to effectively perform the study. All study staffs are trained and routinely re-educated about the ethical conduct of human subject research. There are no anticipated physical, social, legal, or economic risks associated with the study. There is minimal risk of breach of confidentiality. There are no vulnerable populations that are specifically targeted in this study. The findings of this study will be disseminated through peer-reviewed international and national journals, conferences, and seminars.

## Discussion

This study will provide new information to the limited longitudinal data currently available on factors contributing to long-term UI after GDM exposure. It will delineate multiple factors that may help to understand the performance of potential moderator and mediator variables necessary to establish a strategic plan to minimize the underlying mechanisms responsible for increased risk of long-term UI in GDM mothers. An enhanced understanding of the impact of modifiable risk factors can lead to protocols to reduce the rates of UI and associated health care costs, improve the quality of life of affected women and contribute to the global eradication of UI after GDM exposure. It will also enable clinicians to deal more effectively with individual women after their pregnancy and facilitate the transition of the Brazilian Health System from a curative to a preventive strategy [65].

## Conclusion

In conclusion, Diamater is an ongoing study recruiting GDM pregnant women at a prenatal care outpatient clinic to investigate the relationship between GDM plus PS-UI with the occurrence of symptomatic UI plus PFMD 1 year after C-section and assess whether this relation can be explained by hyperglycaemic myopathy. It offers the unique opportunity to establish well-powered biomarker research in a new triad composed of GDM + PS-UI+ hyperglycemic myopathy as a predictor of long-term UI and PFMD. This approach allows for future innovative treatment proposals.

## Supplementary information

**Additional file 1.** Specific protocols for clinical assessment and laboratory analysis of PFM and RAM.

## Data Availability

This is an ongoing study, and data sharing does not apply to this article. No data were generated or analyzed during the current study. Protocol related information can be obtained from the corresponding author (Marilza V.C. Rudge) upon request.

## Abbreviations

ANOVA: One-way analysis of variance
CCL7: Chemokine C-C motif ligand 7
CRF: Case Report Forms
C-section: Cesarean section
DIAMATER: Diabetes and Maternity
ECM: Extracellular Matrix
EMG: Electromyography
EMGs: Surface electromyograms
GAGs: Glycosaminoglycans
GDM: Gestational Diabetes Mellitus
HPLC: High-Performance Liquid Chromatography
ICIQ-SF: International Consultation on Incontinence Questionnaire-Urinary Incontinence-Short Form
IRB: The Institutional Review Board
ISI: Incontinence Severity Index
OGTT: Oral Glucose Tolerance Test
OPLS: Orthogonal partial least squares
PCR: Polymerase chain reaction
PDRC: The Perinatal Diabetes Research Centre
PFM: Pelvic Floor Muscles
PLS: Partial least squares
PS-UI: Pregnancy Specific Urinary Incontinence
PTH: Parathyroid Hormone
RAM: Rectus Abdominis Muscles
RNA: Ribonucleic acid
SENIAM: Surface Electromyography for the Non-Invasive Assessment of Muscles
T2DM: Type 2 Diabetes Mellitus
UI: Urinary Incontinence
UNESP: Universidade Estadual Paulista Júlio de Mesquita Filho

## References

[1] Ajala O, Jensen LA, Ryan E, Chik C (2015). Women with a history of gestational diabetes on long-term follow up have normal vascular function despite more dysglycemia, dyslipidemia and adiposity. Diabetes Res Clin Pract.

[2] Mestman JH, Reece EA, Coustan DR, Gabbe SG (2004). Interaction between pregnancy, gestational diabetes and long-term maternal outcome. Diabetes women adolescence menopause.

[3] Guariguata L, Whiting DR, Hambleton I, Beagley J, Linnenkamp U, Shaw JE (2014). Global estimates of diabetes prevalence for 2013 and projections for 2035. Diabetes Res Clin Pract.

[4] Kim C, McEwen LN, Sarma AV, Piette JD, Herman WH (2008). Stress urinary incontinence in women with a history of gestational diabetes mellitus. J Women’s Heal.

[5] Barbosa AMP, Dias A, Marini G, Calderon IMP, Calderon IMP, Witkin S, Rudge MVC (2011). Urinary incontinence and vaginal squeeze pressure two years post-cesarean delivery in primiparous women with previous gestational diabetes mellitus. Clinics.

[6] Chuang CM, Lin IF, Horng HC, Hsiao YH, Shyu IL, Chou P (2012). The impact of gestational diabetes mellitus on postpartum urinary incontinence: a longitudinal cohort study on singleton pregnancies. BJOG An Int J Obstet Gynaecol.

[7] Kuh D, Hardy R (2002). A life course approach to women’s health.

[8] DeLancey JOL, Kane Low L, Miller JM, Patel DA, Tumbarello JA (2008). Graphic integration of causal factors of pelvic floor disorders: an integrated life span model. Am J Obstet Gynecol.

[9] Saito FH, Damasceno DC, Dallaqua B, Linhares IM, Rudge MV, Calderon IMP (2013). Heat shock protein production and immunity and altered fetal development in diabetic pregnant rats. Cell Stress Chaperones.

[10] Saito FH, Damasceno DC, Kempinas WG, Morceli G, Sinzato YK, Taylor KN (2010). Repercussions of mild diabetes on pregnancy in wistar rats and on the fetal development. Diabetol Metab Syndr.

[11] Kiss ACI, Lima PHO, Sinzato YK, Takaku M, Takeno MA, Rudge MV (2009). Animal models for clinical and gestational diabetes: maternal and fetal outcomes. Diabetol Metab Syndr.

[12] Damasceno DC, Sinzato YK, Bueno A, Netto AO, Dallaqua B, Gallego FQ (2013). Mild diabetes models and their maternal-fetal repercussions. J Diabetes Res.

[13] Damasceno DC, Netto AO, Iessi IL, Gallego FQ, Corvino SB, Dallaqua B (2014). Streptozotocin-induced diabetes models: Pathophysiological mechanisms and fetal outcomes. Biomed Res Int.

[14] Moreli JB, Santos JH, Rocha CR, Damasceno DC, Morceli G, Rudge MV (2014). DNA damage and its cellular response in mother and fetus exposed to hyperglycemic environment. Biomed Res Int.

[15] Boncher N, Vricella G, Kavran M, Xiao N, Hijaz A (2012). Setting a new standard: updating the vaginal distention translational model for stress urinary incontinence. Neurourol Urodyn.

[16] Sapsford R (2004). Rehabilitation of pelvic floor muscles utilizing trunk stabilization. Man Ther.

[17] Heesakkers JPFA, Gerretsen RRR (2004). Urinary incontinence: sphincter functioning from a urological perspective. Digestion.

[18] Yang JM, Yang SH, Yang SY, Yang E, Huang WC, Tzeng CR (2010). Clinical and pathophysiological correlates of the symptom severity of stress urinary incontinence. Int Urogynecol J.

[19] Bakircioglu ME, Sievert KD, Lau A, Lin CS, Lue TF (2000). The effect of pregnancy and delivery on the function and ultrastructure of the rat bladder and urethra. BJU Int.

[20] Li GY, Cui WS, Zhou F, Gao ZZ, Xin H, Liu T (2012). Pathology of urethral fibromuscular system related to parturition-induced stress urinary incontinence and TGF-$β$1/Smad pathway. Mol Cell Biochem.

[21] Mastrocola R, Reffo P, Penna F, Tomasinelli CE, Boccuzzi G, Baccino FM (2008). Muscle wasting in diabetic and in tumor-bearing rats: role of oxidative stress. Free Radic Biol Med.

[22] Chen B, Yeh J (2011). Alterations in connective tissue metabolism in stress incontinence and prolapse. J Urol.

[23] Alperin M, Lawley DM, Esparza MC, Lieber RL (2015). Pregnancy-induced adaptations in the intrinsic structure of rat pelvic floor muscles. Am J Obstet Gynecol.

[24] Alperin M, Kaddis T, Pichika R, Esparza MC, Lieber RL (2016). Pregnancy-induced adaptations in intramuscular extracellular matrix of rat pelvic floor muscles. Am J Obstet Gynecol.

[25] Piculo F, Marini G, Barbosa AMP, Damasceno DC, Matheus SM, Felisbino SL (2014). Urethral striated muscle and extracellular matrix morphological characteristics among mildly diabetic pregnant rats: translational approach. Int Urogynecol J Pelvic Floor Dysfunct.

[26] Marini Gabriela, Piculo Fernanda, Vesentini Giovana, Barbosa Angélica Mércia Pascon, Damasceno Débora Cristina, Matheus Selma Maria Michelin, Hijaz Adonis, Daneshgari Firouz, Rudge Marilza Vieira Cunha (2016). Effects of short-term severe and long-term mild STZ-induced diabetes in urethral tissue of female rats. Neurourology and Urodynamics.

[27] Liu G, Daneshgari F (2006). Temporal diabetes- and diuresis-induced remodeling of the urinary bladder in the rat. Am J Physiol Regul Integr Comp Physiol.

[28] Marini G, Piculo F, Vesentini G, Damasceno DC, Delella FK, Calderon IMP (2018). The influence of hyperglycemia on the remodeling of urethral connective tissue in pregnant rats. Eur J Obstet Gynecol Reprod Biol.

[29] Oishi Y, Ogata T, Yamamoto KI, Terada M, Ohira T, Ohira Y (2008). Cellular adaptations in soleus muscle during recovery after hindlimb unloading. Acta Physiol.

[30] Madill SJ, McLean L (2006). Relationship between abdominal and pelvic floor muscle activation and intravaginal pressure during pelvic floor muscle contractions in healthy continent women. Neurourol Urodyn.

[31] Arab AM, Chehrehrazi M (2011). The response of the abdominal muscles to pelvic floor muscle contraction in women with and without stress urinary incontinence using ultrasound imaging. Neurourol Urodyn.

[32] Vesentini G, Marini G, Piculo F, Damasceno DC, Matheus SMM, Felisbino SL (2018). Morphological changes in rat rectus abdominis muscle induced by diabetes and pregnancy. Brazilian J Med Biol Res.

[33] Liu G, Lin YH, Yamada Y, Daneshgari F (2008). External urethral sphincter activity in diabetic rats. Neurourol Urodyn.

[34] Jia G, Aroor AR, DeMarco VG, Martinez-Lemus LA, Meininger GA, Sowers JR (2015). Vascular stiffness in insulin resistance and obesity. Front Physiol.

[35] Sangsawang B (2014). Risk factors for the development of stress urinary incontinence during pregnancy in primigravidae: a review of the literature. Eur J Obstet Gynecol Reprod Biol.

[36] Melville JL, Katon W, Delaney K, Newton K (2005). Urinary incontinence in US women: a population-based study. Arch Intern Med.

[37] Saydah SH, Chandra A, Eberhardt MS (2005). Pregnancy experience among women with and without gestational diabetes in the U.S., 1995 national survey of family growth. Diabetes Care.

[38] Brown JS, Nyberg LM, Kusek JW, Burgio KL, Diokno AC (2003). Proceedings of the national institute of diabetes and digestive and kidney diseases international symposium on epidemiologic issues in urinary incontinence in women. Am J Obstet Gynecol.

[39] Breitborde NJK, Srihari VH, Pollard JM, Addington DN, Woods SW (2010). Mediators and moderators in early intervention research. Early Interv Psychiatry.

[40] Wong RSM, Yu EYT, Guo VY, Wan EYF, Chin WY, Wong CKH (2018). A prospective cohort study to investigate parental stress and child health in low-income Chinese families: protocol paper. BMJ Open.

[41] Mackinnon D, DP MK (2008). Introduction to statistical mediation analysis.

[42] Baron RM, Kenny DA (1986). The moderator–mediator variable distinction in social psychological research: conceptual, strategic, and statistical considerations. Psycnet Apa Org.

[43] Lenherr SM, Clemens JQ, Braffett BH, Dunn RL, Cleary PA, Kim C (2016). Glycaemic control and risk of incident urinary incontinence in women with type 1 diabetes: results from the diabetes control and complications trial and epidemiology of diabetes interventions and complications (DCCT/EDIC) study. Diabet Med.

[44] Jiménez-Rodríguez J, Carbajal-Ramírez A, Meza-Vázquez H, Moreno-Palacios J, Serrano-Brambila E (2016). Prevalence of urinary tract symptoms in women with diabetes mellitus. Rev Med Inst Mex Seguro Soc.

[45] Isik H, Aynioglu O, Sahbaz A, Selimoglu R, Timur H, Harma M (2016). Are hypertension and diabetes mellitus risk factors for pelvic organ prolapse?. Eur J Obstet Gynecol Reprod Biol.

[46] Rudge Marilza Vieira Cunha, Barbosa Angélica Mercia Pascon, Sobrevia Luis, Gelaleti Rafael Bottaro, Hallur Raghavendra Lakshmana Shetty, Marcondes João Paulo Castro, Salvadori Daisy Maria Fávero, Prudêncio Caroline Baldini, Magalhães Claudia Garcia, Costa Roberto, Abbade Joelcio Francisco, Corrente José Eduardo, Calderon Iracema de Mattos Paranhos (2020). Altered maternal metabolism during mild gestational hyperglycemia as a predictor of adverse perinatal outcomes: A comprehensive analysis. Biochimica et Biophysica Acta (BBA) - Molecular Basis of Disease.

[47] Kraemer HC (2016). Messages for clinicians: moderators and mediators of treatment outcome in randomized clinical trials. Am J Psychiatry.

[48] Kraemer HC, Wilson GT, Fairburn CG, Agras WS (2002). Mediators and moderators of treatment effects in randomized clinical trials. Arch Gen Psychiatry.

[49] Abrams P, Cardozo L, Fall M, Griffiths D, Rosier P, Ulmsten U (2003). The standardisation of terminology in lower urinary tract function: report from the standardisation sub-committee of the international continence society. Urology.

[50] Hvidman L, Hvidman L, Foldspang A, Mommsen S, Bugge NJ (2002). Correlates of urinary incontinence in pregnancy. Int Urogynecol J Pelvic Floor Dysfunct.

[51] Tamanini JT, Dambros M, D'Ancona CA, Palma PC, Rodrigues Netto N (2004). Validation of the “International Consultation on Incontinence Questionnaire -- Short Form” (ICIQ-SF) for Portuguese. Rev Saude Publica.

[52] Pereira VS, Santos JY, Correia GN, Driusso P (2011). Translation and validation into Portuguese of a questionnaire to evaluate the severity of urinary incontinence. Rev Bras Ginecol Obstet.

[53] Atalah ES, Castillo CL, Castro RS, Aldea PA (1997). Propuesta de un nuevo estándar de evaluación nutricional em embarazadas. Rev Med Chile.

[54] Rasmussen KM, Yaktine AL (2009). Weight gain during pregnancy internet.

[55] Messelink B, Benson T, Berghmans B, Bø K, Corcos J, Fowler C (2005). Standardization of terminology of pelvic floor muscle function and dysfunction: report from the pelvic floor clinical assessment group of the international continence society. Neurourol Urodyn.

[56] Liaw LJ, Hsu MJ, Liao CF, Liu MF, Hsu AT (2011). The relationships between inter-recti distance measured by ultrasound imaging and abdominal muscle function in postpartum women: a 6-month follow-up study. J Orthop Sport Phys Ther.

[57] Dietz HP, Shek C, Clarke B (2005). Biometry of the pubovisceral muscle and levator hiatus by three-dimensional pelvic floor ultrasound. Ultrasound Obstet Gynecol.

[58] van Veelen GA, Schweitzer KJ, van der Vaart CH (2014). Ultrasound imaging of the pelvic floor: changes in anatomy during and after first pregnancy. Ultrasound Obstet Gynecol.

[59] Rankin G, Stokes M, Newham DJ (2006). Abdominal muscle size and symmetry in normal subjects. Muscle Nerve.

[60] Koppenhaver SL, Parent EC, Teyhen DS, Hebert JJ, Fritz JM (2009). The effect of averaging multiple trials on measurement error during ultrasound imaging of transversus abdominis and lumbar multifidus muscles in individuals with low back pain. J Orthop Sport Phys Ther..

[61] Hwang H, Bowen BP, Lefort N, Flynn CR, De Filippis EA, Roberts C (2010). Proteomics analysis of human skeletal muscle reveals novel abnormalities in obesity and type 2 diabetes. Diabetes.

[62] Boyle KE, Hwang H, Janssen RC, DeVente JM, Barbour LA, Hernandez TL (2014). Gestational diabetes is characterized by reduced mitochondrial protein expression and altered calcium signaling proteins in skeletal muscle. PLoS One.

[63] Little RJA, Rubin DB (1987). Statistical analysis with missing data.

[64] Worley B, Powers R (2015). Generalized adaptive intelligent binning of multiway data. Chemometr Intell Lab Syst.

[65] Kaiser B, Razurel C, Jeannot E (2013). Impact of health beliefs, social support and self-efficacy on physical activity and dietary habits during the post-partum period after gestational diabetes mellitus: study protocol. BMC Pregnancy Childbirth.

