# Supplementary material for: Study protocol to investigate biomolecular muscle profile as predictors of long-term urinary incontinence in women with gestational diabetes mellitus
Source: BMC Pregnancy Childbirth. 2020 Feb 19;20:117. doi: 10.1186/s12884-020-2749-x (PMC7031907; doi:10.1186/s12884-020-2749-x)
Supplement: Supplementary file 1 — Additional file 1. Specific protocols for clinical assessment and laboratory analysis of PFM and RAM. [file 12884_2020_2749_MOESM1_ESM.docx]

**ADDITIONAL FILE 1.**

**Study protocol to investigate biomolecular muscle profile as predictors of long-term urinary incontinence in women with gestational diabetes mellitus**

**Specific protocols for clinical assessment and laboratory analysis of PFM and RAM**

**Authors:** **Marilza V. C. Rudge,** Fátima P. Souza, Joelcio F. Abbade, Raghavendra L. S. Hallur, João Paulo C. Marcondes, Fernanda Piculo, Gabriela Marini, Giovana Vesentini, Lehana Thabane, Steven S. Witkin, Iracema M. P. Calderon1, Angélica M. P. Barbosa and The Diamater Study Group

**1. Clinical assessment of PFM and RAM**

**1.1 Digital palpation and vaginal squeeze pressure**

For the digital vaginal evaluation, the examiner, using a previously lubricated medical examination glove, introduces her second and third fingers 2-3 cm into the vaginal canal. Next, the participant will be instructed to contract only the pelvic floor muscles, and the intensity of the contraction will be classified as absent, weak, moderate or strong, according to Messelink et al. [1]

Perineometer values higher than 33.6 mmHg will be considered as normal based on our previous nulliparous women study. The perineometer used will be selected based on Barbosa’s study [2].

First, vaginal bidigital palpation will be performed with the woman’s knees semi flexed. The subjects will be asked to firmly grasp their vagina three times. After this, the women will stay in the same position, and a vaginal latex sensor called a Perinaperineometer will be employed to measure the strength of the grasps (Quark, São Paulo, Brazil).

Perineometry will be conducted using a PerinaStimperineometer (Quarck, Piracicaba, São Paulo, Brazil). This device consists of a vaginal probe covered with a thick latex sheath held in place by rubber bands. A non-lubricated condom will be placed over the probe, which will then be introduced into the vagina and gently inflated until the patient reports feeling the contact of the probe against the vaginal wall and before she reports any sensation of pain. Next, the evaluator presses the “zero” key to reset the device, and variations in PFM pressure are displayed on a linear pressure scale represented by a luminous light-emitting diode (LED) scale.

**1.2. The 3D pelvic floor ultrasound (PFUS)**

Levator function will be assessed using the modified Oxford grading of muscle strength scoring system^14^. Transperineal ultrasonographic assessment biometry of PFM and RAM data will be collected from all pregnant women at T1, T2, T4, and T6 waves including anteroposterior diameter, transversal diameter, Hiatal area, and levator ani muscle thickness.

PFUS will be performed with the woman in the lithotomy position after voiding, at rest after bladder emptying using GE Voluson “i” system with RAB 2-6 RS (2-6 MHz) curved array three-dimensional transducer (GE Healthcare, Zipf, Austria) with an 8-4-MHz curved-array volume transducer at an acquisition angle of 70° in the sagittal plane and 85° in the coronal plane (frame rate is approximate 3 Hz) [3,4].

Volume achievement will be accomplished at rest, on maximum Valsalva maneuver, and maximum pelvic floor muscle contraction (PFMC).

Each patient will perform at least three Valsalva maneuvers, and volume data demonstrating the greatest degree of pelvic organ descent will be used for analysis. Offline analysis of the rendered volume datasets will be conducted after collecting all patients’ data, using the 4D View – version 14 Ext 3 (GE Healthcare) software program. All datasets will be analyzed in random order by one specialist from our group who will be blinded to all patient data [5].

**1.3. The 3D RAM ultrasound protocol**

First, the transducer will be placed above the umbilicus. It will be moved laterally from the midline until the cross-section of the muscle will center on the image [6]. Ultrasound images will be acquired in the B-mode using a portable ultrasound unit, the GE Voluson “i” system with a RAB 2-6 RS (2-6 MHz) curved array three-dimensional transducer (GE Healthcare, Zipf, Austria). The thickness of the lateral abdominal muscles will be assessed at rest and during physical tasks that required a submaximal contraction of these muscles. In addition, measurements of the thickness and cross-sectional area of the rectus abdominals muscles will be obtained at rest. To help avoid an order effect, the images will be obtained in a counterbalanced order. Three images of the resting and contracting states will be obtained bilaterally for a total of 36 images analyzed for each participant [7].

The anatomic, functional, and diastasis evaluation of RAM by 2D US [8-13] will be conducted with the woman in the dorsal decubitus position, and lower members extended. The two-dimensional (2D) ultrasound (US) evaluation will be performed by a physician or physiotherapist with specific training on the image capturing and measuring. In order to obtain good compliance, all examinations will be accompanied by the PI.

The anatomic and functional evaluation will be conducted in the lower right portion of RAM in a transversal direction along the muscle [14]. A different area will be selected for diastasis evaluation to avoid bias. A linear transductor (8 to 12 MHz) will be placed transversely 2 cm above and to the right of the upper ridge of the pubic symphysis. The anatomic variables anteroposterior and transversal thickness of RAM (right portion) will be acquired at rest. For RAM functional evaluation the anteroposterior and transversal thickness acquired with PFM under sustained contraction will be analyzed, with RAM under sustained contraction and upper body lifted, and under the PFM co-contraction.

PFM contraction will be done to evaluate RAM response in front of such stimuli. However, before RAM image acquisition under PFM contraction, the woman will be submitted to PFM function according to the International Continence Society [1], to avoid bias during US evaluation and to analyze its function subjectively.

For RAM diastasis evaluation [12,13], the transductor will be placed transversely along the midline of the abdomen, 2 cm above and 2 cm below the center of the umbilicus, respectively.^15^ The diastasis referent measures considered will be those less than 1.5 cm at the xiphoid appendix, 2.2 to 3 cm above and 0.6 cm to 2 cm below the center of the umbilicus, according to previous works [5,16-19]. All acquired images will be coded and stored for further off-line blinded analysis using the software.

**1.4. Electromyography**

Surface electromyography (EMGs) obtains knowledge of the neuromotor behaviour of a muscular group, recording behaviour of muscle fibers by simultaneous potential actions during resting or function. The research will include the behaviour of four muscles during a function that increases intra-abdominal pressure and the interaction of these four muscles during pelvic floor contraction. We choose rectus abdominis (1/2 distance between pubis and umbilicus and approximately 3 cm lateral to the midline), transverses/obliquus internus (2 centimetres medial and inferior to the anterior superior iliac spine (ASIS)), a deep (vaginal probe sensor will be positioned on both vagina wall sides) and superficial (bipolar electrode in perineal region above the anal border) portion of pelvic floor because they play an important role incontinence.

EMGs will be recording by using an eight-channel device (New MiotoolUro Wireless; Porto Alegre, Brazil) 16-bit A/D converter, a sampling frequency rate fixed at 2000 Hz, automatic gain, safety isolation for 3000 volts and common mode rate (CMRR) of 126 db. The signal will be filtered using a 20-500 Hz dual-pass Butterworth second-order digital filter. The preparation of skin, sensor placement, and location preparation will be performed according to the recommendation of the Surface Electromyography for the Non-Invasive Assessment of Muscles (SENIAM). Modified Glazer protocol will be used to verify muscle activity during rest-activity and fast and hold contractions. These protocols will be used twice, one only asking to increase abdominal pressure without conscious contraction of pelvic floor and the second time only performing pelvic floor contractions.

The raw signal will be processed using Miotec Suite software by an examiner blinded to the women’s clinical data. The electrical data of the recruitment root mean square (RMS) from the period of rest-activity will be obtained by using Hanning window processing of the duration of the rest-activity period. The five fast and five hold contractions will be performed by using Hanning window processing and selecting the most stable period. Calculations of each RMS arithmetic mean of the fast and hold contractions will be performed to determine a single mean value for each contraction type. To normalize the EMG recruitment signal, we will use the maximal fast contraction amplitude (RMS) chosen from among the five fast contraction values at 24–30 weeks of gestation because that will constitute base data for analysis of changes in PFM activity.

**Laboratory data**

The serum concentration of CCL7, relaxin, calcium, parathyroid hormone, Calcitonin, Vitamin D, and insulin will be determined in all participants of the study after fasting for at least 8 hours.

**Maternal blood collection and analysis for biochemical analysis**: The biological material for analysis of the levels of relaxin, insulin, glucose, CCL7, Calcium, calcitonin, PTH, and vitamin D will be maternal blood. Eight mL of blood will be collected with a disposable syringe. After clot formation and centrifugation, the serum will be stored in aliquots at -80 °C for further batch processing according to the specific assay kit. The PTH (pg / mL), calcitonin (pg / mL), vitamin D (mg/dL), glucose (mg/dL) and insulin (pU / mL) analyses will occur simultaneously to the steps and will be performed at the University Hospital-UNESP. All analyses will be in duplicate, using ELISA tests, and protocols will follow instructions contained in the manufacturer's manual, R&D Systems Catalog Number DRL200, R&D Systems Catalog Number DINS00, and R&D Systems Catalog Number DCC700. The analysis of the total calcium levels will be performed in duplicate, using a colorimetric test (Catalog No. 448), calcitonin and PTH by the chemiluminescence method, vitamin D by high-performance liquid chromatography (HPLC) following the procedures of processing and analysis in the manufacturer's manual

**2. RAM sample collection and laboratory assays**

**2.1. The biological material of rectus abdominal muscle (RAM)**

Tissue samples of RAM from the region of the transverse Pfannenstiel incision will be obtained during C-section, after repair of the uterus and serosal edges overlying the uterus and bladder issue and closure of the parietal peritoneum. RAMs are of mixed muscle fiber type [20,21]. A 4 cm RAM tissue aliquot will be sampled from the lower portion, dissections of skeletal muscle will be obtained within 10 min of delivery, dissected free from visible adipose and connective tissues, immediately divided into 6 fragments and stored according to different assays for further analysis.

**2.2. Morphological assays**

Part of the RAM will be harvested, powdered with talcum, frozen in liquid nitrogen and maintained at -80 °C until processed by histochemical and Immunohistochemical techniques for morphometric analysis and, to analyze the distribution, quantification and characterization of the key structural ECM components, such as type I and III collagens, the collagen type I/III ratio and glycosaminoglycans (GAGs). Cross-sections of the samples will be examined via light microscopy and photographed. The morphometric analyses will be performed with Image-Pro Plus software (Version 7.0, Media Cybernetics, Silver Spring, MD) at Case Western Reserve University (OH). Proportions of the RAM that fit each of the following types will be recorded: striated muscle, collagen, and blood vessel. Other samples (three samples/group) will be immersed in a fixative solution containing 0.1% ruthenium red, 3% glutaraldehyde, and 0.1 M cacodylate buffer for 12 h at 4 °C prior to the usual procedures for transmission electron microscopy will be applied for the ultrastructural analyses of RAM.

**3. Molecular analysis of muscle fiber function and ECM-related genes and proteins**

**3.1. Gene expression (PCR ARRAYS®)**

After collection, approximately 100 mg of RAM will be placed in RNA later solution and kept at 4 °C for 24h. The solution will then be removed and the cryotube containing the RAM stored at -80 °C until processing for gene expression analysis. The extraction of total RNA will be performed using TRIzol® reagent (Life Technologies, Carlsbad, CA), according to the manufacturer’s instructions. The analysis of 12 genes of interest (MHCII a: intermediary fibers; MCHII x/d: glycolytic fast fibers; COL1A2: Collagen I; COL3A1: Collagen III; MMP1/ MMP2/ MMP3/ MMP9: Matrix metallopeptidase 1-9; and TIMP1/ TIMP2/ TIMP3: TIMP metallopeptidase inhibitor 1-3) will be carried out using customized PCR Arrays® plates (RT2 Profiler PCR Array - Qiagen). The RNA will be reverse transcribed using the RT2 First Strand kit (SA Biosciences), according to the manufacturer’s protocol. An aliquot of the diluted first-strand synthesis reaction will then be added to the SYBR Green/ROX master mix (SA Biosciences) along with nuclease-free water in accordance with the PCR array system’s user manual. Subsequently, 25 uL of this solution will be placed in each well of the PCR array plate. The plate will be sealed and transferred to the thermocycler 7500 Fast Real-Time PCR equipment (Applied Biosystems). The cycling profile will be described by the manufacturer´s instructions, and the analysis of our results will be done using the ΔΔCT method. The CT values will be calculated, and melting curves constructed. All of the arrays will be performed in triplicate. Analyses of significant gene clusters will be performed using bioinformatics tools available at <http://pcrdataanalysis.sabiosciences.com/pcr/arrayanalysis.php>.

**3.2. Protein expression (Western Blot)**

After collection, approximately 100 mg of RAM will be frozen in liquid nitrogen, stored at -80 °C and used for subsequent analysis of MHCI (slow fibers), MHCIIa (intermediary fibers), MCHII x/d (glycolytic fast fibers), COL1A2 (Collagen I), and COL3A1 (Collagen III) protein expression by Western Blot. RAM samples will be homogenized using the TURRAX – Politronhomogenator (3 cycles of 5 sec) in extraction buffer containing 50 mM Tris-HCl pH 7.5, 0.2M NaCl, 0,25% Triton X-100, 2mM EDTA, and 1% protease inhibitors (Sigma Co, Saint Louis, MO, USA), incubated at 4°C and centrifuged at 12.000 RPM for 15 min. at 4 °C. The supernatant will be used for subsequent steps, including protein quantification by Bradford method (1976), electrophoresis of protein extracts (40 pg) in 8% polyacrylamide gels and its transfection to Hybond ECL nitrocellulose membranes (Amersham, Little Chalfont, Reino Unido), incubation (1h) with blocking solution (5% skim milk in Tris Buffered Saline with Tween® 20), followed by incubation with primary (12h at 4°C) and secondary (30 min. at 37°C) specific antibodies and wash steps (in PBS) after each incubation step. The immunoreactive components will be revealed by incubation (5 min at 37°C, protected from light) in a solution containing 0.6% DAB (diaminobenzidine; Sigma, USA), 0.06% hydrogen peroxide, 1% DMSO in phosphate buffer solution. The bands will be analyzed and quantified by densitometry and normalized to β-actin expression.

**3.3 Transcriptomics, metabolomics, and proteomics**

**3.3.1. Transcriptomics**

After collection, approximately 100 mg of RAM will be placed in RNA later solution and kept at 4 °C for 24h. The RNA later solution will then be removed, and RAM stored at -80 °C until processed by the RNA-seq technique. The extraction of total RNA will be performed using TRIzol® reagent (Life Technologies, Carlsbad, CA). The samples with an RNA integrity number (RIN) > 7 will be used for RNA sequencing.

To construct the sequencing libraries amplified cDNAs with kit (FC-131-1024, illumine) will be used, also using the HiSeq2500 system in the 100-bp paired-end mode of the TruSeq Rapid PE Cluster kit and the TruSeq Rapid SBS kit. The software fastqc63, FASTQ Quality Filter, FASTA/Q Clipper will be used to identify and remove the low-quality adapters and reads. Relative gene expression will be quantified by Deseq algorithm, evaluable in Bioconductor/R64 packages. Gene Ontology analysis will be performed for function determination of differentially expressed genes. Genes related to muscle phenotype and those related to muscle alterations promoted by gestational hyperglycemic myopathy will be selected for qPCR validation.

**3.3.2. Metabolomics analysis by nuclear magnetic resonance (M-NMR)**

Metabolomics will be performed to map and to identify the metabolites present, making it possible to understand the dynamic aspects of the metabolic response and identify molecular biomarkers [22-26]. The samples collected will be frozen using liquid nitrogen. The sample, 100 mg of frozen tissue, will be processed, lyophilized, and stored at –80 °C for further NMR analysis [27]. The lyophilized sample will be resuspended in 500 μL D_2_O, containing 3.0 mM 2,2-dimethyl-2-silapentane-5-sulfonate (DSS) to refer the chemical shift (at δ = 0.00) of the NMR spectrum. The final volume of the sample will be adjusted to 600 μL using D2O with 25 mM DSS and transferred to a 5mm NMR tube 70. ^1^H NMR data acquisition will be performed using the Bruker Avance III HD NMR, equipped with cryogenically cooled Z-gradient probes operating at 1H frequencies of 600 MHz (BrukerBioSpin GmbH, Germany). The experiments will be performed using the pulse sequence NOESYGPPRD1D with a mixing time of 100 ms. The spectra will be collected, and processing as phase and baseline correction will be performed using the TopSpin software version 3.2.

Multivariate analyses of the NMR data will be carried out by PCA (main Component Analysis) or multivariate methods of supervised statistics (PLS or OPLS), aiming to build models for Brazil of the samples and thus extract metabolic signatures from specific groups [28]. The calculations will be made by written (homemade) programs by our research group using the MATLAB platform (MathWorks, USA). The scoring and loading charts of the PCA analysis will be used for the display of the data. In the score graph, each point represents an NMR spectrum (i.e. a sample) for one of the main components, and the loading graph visualizes the contribution of key metabolites (statistically significant variables) to the main component. The PCA method will be applied initially to the complete set of data. Then the multivariate supervised methods (PLS or OPLS) will be employed with a prior differentiation of the sampling groups.

**3.3.3. Proteomics**

After collection, approximately 100 mg of RAM will be frozen in liquid nitrogen, stored at -80 °C and used for proteomic analysis. RAM samples will be homogenized in extraction buffer containing 1% protease inhibitors (Sigma Co, Saint Louis, MO, USA), and centrifuged at 10,000g by 20 min at 4°C. The supernatant will be filtered, and the filter-retained material (> 10 kDa) will be collected, lyophilized and stored at -80°C until mass spectrometry analysis. A total of 100 pg of protein extract will be analyzed using a mass spectrometer equipped with an ESI source and a hybrid analyzer LCMS-IT-TOF system (Shimadzu). Data will be collected at scan ranges of 50-3000 m/z, with a resolution of 15.000 m/z, approximately. Protein identification and quantification will be done by LCMS Solution software (v3.5 – Shimadzu), ProteinL Layer (Shimadzu) software, and the Mascot search engine (v2.3, Matrix Science Ltd.) using the Swiss-Prot database (UniProtKB). The Scaffold (v2.04.00) will be used for protein validation. The quantitative proteomics analysis will be carried out using a label-free method.

The MALDI imaging technique will be applied to determine the distribution of the most important proteins on the 15 pm histological muscle slides. Protein digestion by trypsin deposition (20 mg/mL trypsin in 20 mM NH4HCO3 buffer, pH 8.0) will be performed by a chemical inkjet printer (ChIP-1000, Shimadzu) directly on the muscle slides, and incubation in a humid chamber at 37 °C. The tryptic peptides will be analyzed using a matrix solution containing 10 mg/mL cinnamic acid, 50% (V/V) acetonitrile, and 0.1% (v/v) TFA. Protein determination and quantification will be performed using the MALDI ToF-ToF (AXIMA Performance, Shimadzu). The Mascot search engine (v2.3, Matrix Science Ltd.) will be used for protein identification. After the identification of interested proteins, RX/RMN diffraction crystallography will be performed to determine the protein tridimensional structure.

**3.4. Ex-vivo assessment of RAM contractility**

Intact RAM will be isolated, and the contractile portions of the muscle with and without hyperglycemic myopathy will be established. The experimental protocol will measure RAM contractility, corresponding to a mostly slow-oxidative (type I and IIa muscle fibers) and a mostly fast-glycolytic muscle (type IIb and IIx muscle fibers) with distinct contractile properties.

In this protocol, the muscle sample of 1 cm^2^ (n = 20/group) will be isolated and immersed in PBS at 4 °C, gently coupled to integrated platinum electrodes, the chambers of the Myograph equipment (Model 820MS; Danish Myo Technology®) submerged in Krebs physiological solution [composition in mM: NaCl 118.5, 4.7 KCl, NaHCO_3_ 25, 1.2 MgSO_4_, 1.2 KH_2_PO_4_, 2.5 CaCl_2_, 5.5 D-glucose, 300 pM L-arginine, pH 7.4], kept at 37 °C and constantly bubbled with 95% O_2_ /5% CO_2_ mixture for isometric force measurements. The maximum contraction stimulus will be performed by adjusting the voltage to obtain the maximum tensile force and subtracting 20% of the maximum tension to obtain the submaximal stimulus. Changes in isometric tensile strength will be recorded using LabChart software (LabChart 7 for Windows, ADInstruments) coupled to PowerLab (PowerLab Data Acquisition System, ADIntruments) software for capturing and storing muscle responses. The muscular mechanical responses will be expressed as a percentage of the maximum contraction induced by electric stimulation (Grass Model S48). The combination of this type of study plus genetic approaches and biochemical analyses can provide important information on the mechanisms of contractile alteration in skeletal muscle with diabetic myopathy [29].

**References**

1. Messelink B, Benson T, Berghmans B, Bø K, Corcos J, Fowler C, et al. Standardization of terminology of pelvic floor muscle function and dysfunction: report from the pelvic floor clinical assessment group of the International Continence Society. Neurourol Urodyn 2005;24:374–80.
2. Barbosa AMP, Dias A, Marini G, Calderon IMP, Witkin S, Rudge MVC. Urinary incontinence and vaginal squeeze pressure two years post-cesarean delivery in primiparous women with previous gestational diabetes mellitus. Clinics 2011;66:1341-5.
3. Dietz HP, Shek C, Clarke B. Biometry of the pubovisceral muscle and levator hiatus by three-dimensional pelvic floor ultrasound. Ultrasound Obstet Gynecol 2005;25:580-5.
4. van Veelen GA, Schweitzer KJ, van der Vaart CH. Ultrasound imaging of the pelvic floor: changes in anatomy during and after first pregnancy. Ultrasound Obstet Gynecol 2014;44:476-80.
5. Lemos A, Melo EFJ, Dornelas de Andrade A. Respiratory muscle force assessment in the final three months of pregnancy. Brazilian J Phys Ther 2005;9:151-6.
6. Rankin G, Stokes M, Newham DJ. Abdominal muscle size and symmetry in normal subjects. Muscle Nerve 2006;34:320-6.
7. Koppenhaver, SL, Parent, EC, Teyhen, DS, Hebert JJ, Fritz JM. The Effect of averaging multiple trials on measurement error during ultrasound imaging of transversus abdominis and lumbar multifidus muscles in individuals with low back pain. J Orthop Sport Phys Ther 2009;39:604-11.
8. Dietz HP, Haylen BT, Broome J. Ultrasound in the quantification of female pelvic organ prolapse. Ultrasound Obstet Gynecol 2001;18:511-4.
9. Dietz HP. Levator function before and after childbirth. Aust New Zeal J Obstet Gynaecol 2004;44:19-23.
10. Aukee P, Usenius JP, Kirkinen P. An evaluation of pelvic floor anatomy and function by MRI. Eur J Obstet Gynecol Reprod Biol 2004;112:84–8.
11. Hoyte, L, Schierlitz, L, Zou, K, Flesh G, Fielding JR. Two- and 3-dimensional MRI comparison of levator ani structure, volume, and integrity in women with stress incontinence and prolapse. Am J Obstet Gynecol 2001;185:11-9.
12. Mendes, DA, Nahas, FX, Veiga DF, Mendes FV, Figueiras RG, Gomes HC. et al. Ultrasonography for measuring rectus abdominis muscles diastasis. Acta Cir Bras 2007;22:182-6.
13. van Uchelen JH, Kon M., Werker PM. The long-term durability of plication of the anterior rectus sheath assessed by ultrasonography. Plast Reconstr Surg 2001;107:1578–84.
14. Liaw LJ, Hsu MJ, Liao CF, Liu MF, Hsu AT. The Relationships Between Inter-recti distance measured by ultrasound imaging and abdominal muscle function in postpartum women: a 6-month follow-up study. Journal of Orthopaedic & Sports Physical Therapy, 2011;41(6), 435–443.
15. Sancho MF, Pascoal AG, Mota P, Bø K. Abdominal exercises affect inter-rectus distance in postpartum women: a two-dimensional ultrasound study. Physiotherapy 2015;101:286-91.
16. Gilleard WL, Brown JM. Structure and function of the abdominal muscles in primigravid subjects during pregnancy and the immediate postbirth period. Phys Ther 1996;76:750–62.
17. Mesquita LA, Machado AV, Andrade AV. Fisioterapia para redução da diástase dos músculos retos abdominais no pós-parto. Rev Bras Ginecol e Obs 1999;21:267-72.
18. Bursch SG. Interrater reliability of diastasis recti abdominis measurement. Phys Ther 1987;67:1077–9.
19. Rath AM, Attali P, Dumas JL, Goldlust D, Zhang J, Chevrel JP. The abdominal linea alba: an anatomo-radiologic and biomechanical study. Surg Radiol Anat 1996;18:281–8.
20. Hwang H, Bowen BP, Lefort N, Flynn CR, De Filippis EA, Roberts C. et al. Proteomics analysis of human skeletal muscle reveals novel abnormalities in obesity and type 2 diabetes. Diabetes 2010;59:33-42.
21. Boyle KE, Hwang H, Janssen RC, DeVente JM, Barbour LA, Hernandez TL et al. Gestational diabetes is characterized by reduced mitochondrial protein expression and altered calcium signaling proteins in skeletal muscle. PLoSOne 2014;9:e106872.
22. Sussulini A, Prando A, Maretto DA, Poppi RJ, Tasic L, Banzato CE. et al. Metabolic profiling of human blood serum from treated patients with bipolar disorder employing ^1^H NMR spectroscopy and chemometrics. Anal Chem 2009;81:9755-63.
23. Sumner LW, Amberg A, Barrett D, Beale MH, Beger R, Daykin CA. et al. Proposed minimum reporting standards for chemical analysis. Metabolomics 2007;3:211-221.
24. Griffin JL, Des Rosiers C. Applications of metabolomics and proteomics to the mdx mouse model of Duchenne muscular dystrophy: lessons from downstream of the transcriptome. Genome Med 2009;1:32.
25. Griffin JL. Understanding mouse models of disease through metabolomics. Curr Opin Chem Biol 2006;10:309-15.
26. Schripsema J. Application of NMR in plant metabolomics: techniques, problems and prospects. Phytochem Anal 2010;21:14-21.
27. Wishart DS, Tzur D, Knox C, Eisner R, Guo AC, Young N. et al. HMDB: the human metabolome database. Nucleic Acids Res 2007;35:D521-6.
28. Worley B, Powers R. Generalized adaptive intelligent binning of multiway data. Chemometr Intell Lab Syst. 2015;146; 42–6.
29. Park KH, Brotto L, Lehoang O, Brotto M, Ma J, Zhao X. Ex Vivo Assessment of contractility, fatigability and alternans in isolated skeletal muscles. J Vis Exp 2012;69:e4198.
